# The Opposition to the State Faculty Bill

**Published:** 1884-02

**Authors:** 


### THE OPPOSITION TO THE STATE FACULTY BILL.

"THE WORLD'S DISPENSARY" OF BUFFALO, THE BUFFALO MEDICAL COLLEGE, WARNER'S SAFE REMEDY COMPANY OF ROCHESTER AND THE BELLEVUE MEDICAL COLLEGE OF NEW YORK LEAD THE OPPOSING FORCES.

On Wednesday, the 23d ult., the joint committee of the Senate and Assembly gave a hearing to the opponents of the State

Faculty Bill, who were present in force and presented their case with so much eloquence and energy that the hours between 3.00 in the afternoon and 11.30 in the evening were consumed in the hearing. The advocates of the bill had previously presented their case, but were also invited to be present on this occasion, and some of them offered a few remarks. But it is to the motley crowd composing the opposition to the bill to which we would pay particular attention at this time; and we need only say that the advocates of the bill dignifiedly presented the plain and unanswerable facts and arguments which have again and again appeared in the JOURNAL, and which not only the profession but intelligent men of every class everywhere recognize as affording abundant reason for the passage of the bill.

Its supporters worthily represented the medical men of the State, and were fitly led by the Nestor of the profession, Edward M. Moore, M. D., of Rochester. At the hearing the Legislative Committee decided that the opponents of the bill should first be heard, and the friends of the measure should close the debate. We have not time to dwell upon it, but our readers will not need to be told that not only the argument but also the presentation of it was in delightful contrast.

Let us now turn our attention to the consideration of the opposition.

First, as to the opponents.

In almost every instance, men were there as representatives of institutions or societies, and the principal institutions arrayed against the bill were the World's Dispensary Association of Buffalo, by its President, Hon. Ray V. Pierce; the Medical Department of the University of Buffalo, by its attorney, Francis Lynde Stetson, Esq., of New York; Warner's Safe Remedy Co., of Rochester, by Dr. Henion; the United States Medical College of New York, by Dr. Robert A. Gunn, and Bellevue Medical College, by Professor Austin Flint, *Junior*. There were some other corporations present, but none of them took equal prominence or consumed as much time as those we have cited. There are doubtless those in the profession who would deem this a strangely assorted company, who would be inclined to doubt their having any common interest or ground from which to oppose a bill supported by the medical profession of the State; but we are well informed that the arguments of the representatives of the different corporations were well nigh identical; there was the one refrain to be heard in each and all: We are manufacturers. Our factories are ample; our products please us and please the people. The demand for them is constantly increasing. We are the natural and the best judges of the superiority of our own productions, and we wish to be let alone. There were many attempts at variations of this discourse; there were appeals to past services, to ancestral advantages, to the shortcomings of similar factories in other places, but the variation soon ended in the refrain, we are very fond of ourselves and we wish to be let alone.

An unbiased layman, who heard the speeches, said to us that the opponents of the bill, by their own arguments, did more to strengthen the measure than even its own advocates, and that unless more convincing reasons were to be offered, the passage of the bill could be safely predicted.

But one more thought upon the opposition to the bill and we close.

To whom do the members of the medical faculties opposing this bill owe professional allegiance? At a remarkably full meeting of the Medical Society of the County of Erie, the bill is endorsed unanimously, but the fact seems to have no weight with the resident professors of one of our colleges. The medical societies of New York and Kings counties give the measure similar approval, and the fact is, in like manner, ignored by the men who grind at the larger mill. In conclusion, we again ask the question: To whom do the managers of these corporations owe allegiance?

Is it possible that personal selfishness and the desire for "filthy lucre" can stifle all nobler aspirations and sense of higher duties?

